# Synchronous Testicular Cancer in Monozygotic Twins

**DOI:** 10.7759/cureus.22956

**Published:** 2022-03-08

**Authors:** Ioannis Manolitsis, Lazaros Tzelves, Themistoklis Bellos, Marinos Berdempes, Andreas Skolarikos

**Affiliations:** 1 Second Department of Urology, National and Kapodistrian University of Athens, Sismanogleio General Hospital, Athens, GRC

**Keywords:** familial testicular cancer, cryptorchidism, monozygotic twins, metastatic testicular cancer, testis

## Abstract

Testicular cancer is the most common neoplasm in men aged 15-45 years old, with several established risk factors such as cryptorchidism, age, and family history. We report a case of a 44-year-old man with a history of cryptorchidism who presented with lesions in his right testis, a large retroperitoneal mass, and diffuse nodal lesions in his lungs. He underwent radical orchiectomy that showed testicular cancer and was immediately inducted into systemic therapy based on bleomycin, etoposide, and cisplatin. Two weeks later, his monozygotic twin brother, also reporting cryptorchidism at a young age, presented with a painless mass in his left testis. He underwent radical orchiectomy that revealed testicular cancer and received adjuvant chemotherapy. The first patient, after two cycles of chemotherapy, suffered from an episode of massive hematochezia and died while his brother remains relapse-free.

## Introduction

Testicular cancer (TC) is a rare entity, accounting for 1% of all adult neoplasms. Despite that, it is the most common cancer and the leading cause of cancer-related mortality and morbidity among males aged 15-45 years old [1-2]. The main histological type of TC is germ cell tumors (GCT) (90-95% of cases) [3]. TC germ cell tumors are divided into seminomas and non-seminomas, each contributing to 50% of all cases. Non-seminomas include several histological subtypes (embryonal cell carcinoma, yolk sac tumor, choriocarcinoma, teratoma) [3]. Several risk factors for TC have been identified. TC affects mainly white Caucasian populations of a young age [4]. The strongest risk factor is cryptorchidism or maldescended testis [5]. Other risk factors include occupational exposure (firefighters), exposure to organochlorine pesticides, disorders of sexual development, fertility disorders, viral infections (Epstein-Barr virus, cytomegalovirus, B19 parvovirus, and human immunodeficiency virus), testicular carcinoma in situ, and previous TC history [6]. There is evidence that supports inherited susceptibility for testicular cancer, with the risk being fourfold higher in brothers and twofold higher in sons of men with a history of TC [7]. We report here a case of monozygotic twin brothers who presented with synchronous testicular cancer.

## Case presentation

**Figure 1:**
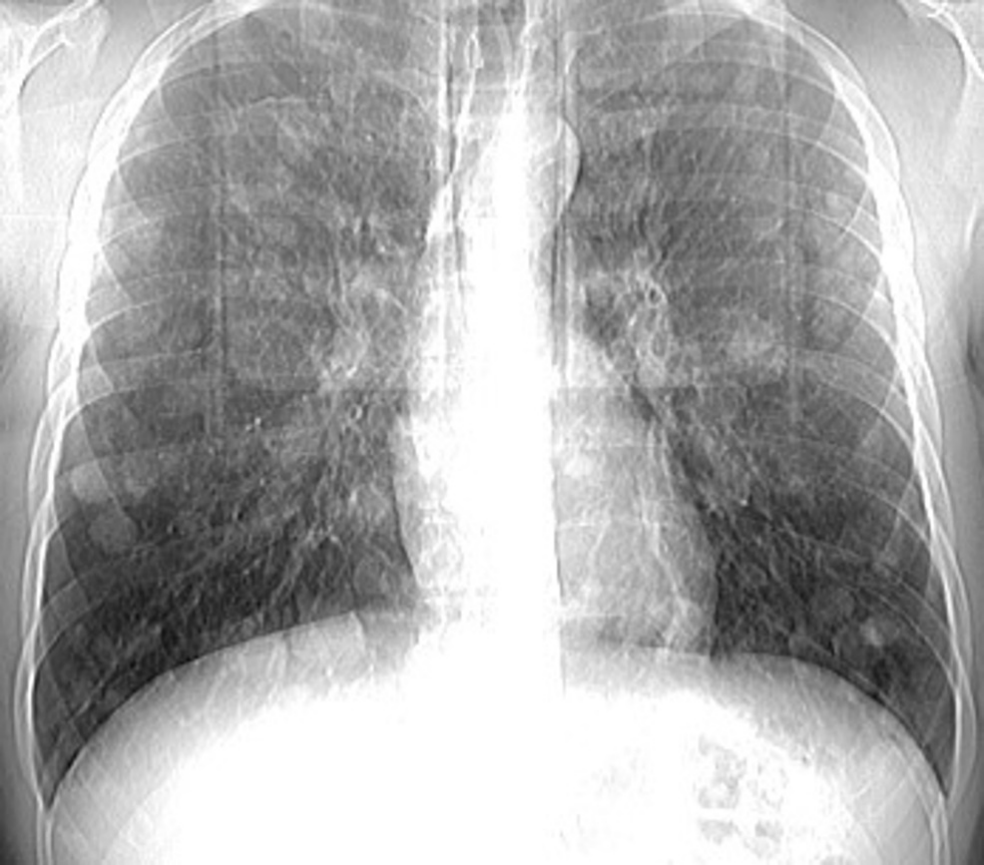
Diffuse nodal lesions in all lung fields

**Figure 2:**
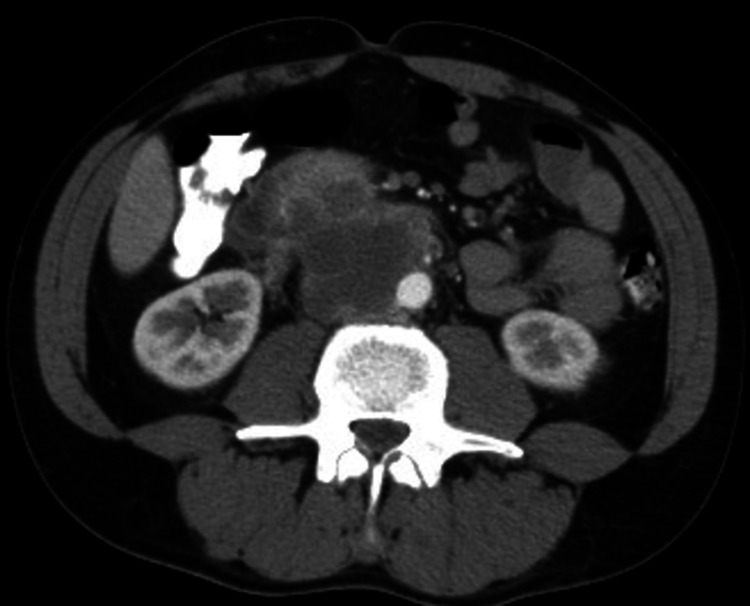
Contrast-enhanced CT scan of the abdomen revealing a large retroperitoneal mass

A 44-year-old man presented to the emergency department reporting abdominal pain. Physical examination showed abdominal tenderness, and a suspicious finding during palpation of the right testis. The patient reported cryptorchidism of his left testis, for which he underwent orchiopexy in his adolescence. He underwent a full panel of blood tests and imaging studies. The chest x-ray showed diffuse nodular lesions in all lung fields (Figure 1). The CT scan revealed a large retroperitoneal mass featuring areas of central necrosis (Figure 2). He consequently had a scrotal ultrasound that showed four hypoechoic lesions in the right testis (4.5-4-3, 2-2 cm). The blood tests revealed a normal blood count with increased lactate dehydrogenase (LDH), alpha-fetoprotein (AFP) levels (100 ng/mL), and beta-human chorionic gonadotropin (β-hCG) levels of 20.000 mIU/mL (reference values: 0-5). The patient was counseled to visit a sperm bank for sperm cryopreservation and afterward, a right radical inguinal orchiectomy was performed. The pathology report showed seminoma staged T1a according to the current TNM (tumor, node, metastasis) classification [8], mature teratomas, and lesions of germ cell neoplasia in situ. Immunochemistry revealed CD117+, CD30-, HCG-, Oct3/4+, and PLAP+. The patient was then immediately referred to the oncology department due to the high metastatic burden, where he proceeded with systemic treatment based on bleomycin, etoposide, and cisplatin (BEP), scheduled initially for four cycles.

Two weeks later, his monozygotic twin brother presented to our department with a painless mass in his left testis, after being advised to undergo screening. He also reported cryptorchidism of his contralateral testis operated (orchiopexy) at the age of two. He underwent a complete blood count and biochemical profile, with the serum markers AFP, β-hCG, and LDH all being within normal range. The scanning of his thorax showed no abnormalities, and the MRI of his scrotum revealed a mass of 4.5 cm in size, located in the upper pole of his left testis. The patient consequently underwent left radical inguinal orchiectomy, with the pathology report showing a testicular seminoma staged T1b with rete testis invasion. The immunochemistry showed CD117+, Oct3/4+, and AFP-, and the patient was inducted into adjuvant chemotherapy protocol based on one cycle of BEP, after being advised that both active surveillance and adjuvant chemotherapy are viable treatment options [8].

A month later, the first patient, after completing two cycles of chemotherapy with objective clinical, biochemical, and imaging improvement in all metastatic lesions (thorax, retroperitoneum), suffered from an episode of massive hematochezia and died. The second patient remains relapse-free and on a close surveillance protocol.

## Discussion

Testicular cancer is the most common cancer in men aged 15-45 years old. The etiology still remains unclear, even though several risk factors have been established, such as cryptorchidism, age, and family history [5]. Compared to the general population, the risk of testicular cancer appears to be higher in siblings and sons of affected men [6]. There have been migration studies in the Nordic countries supporting the hypothesis of genes contributing to testicular cancer rather than environmental exposures [9]. A study by Greene et al. has proposed the existence of an autosomal recessive model of inheritance for familial TC and identified genomic regions such as Y chromosome microdeletions and PDE11A gene mutations of chromosome 2 as testicular germ cell tumor risk factors [10]. Familial association of TC constitutes around 2% of all cases, with the risk of TC being three-to-six times higher when a brother had a history of TC [7]. It has been shown that there is a tendency toward a close age at TC diagnosis between relatives and that could be explained due to either concordant exposure to environmental risk factors, or genetic determinants of the age of cancer development in a family or a combination of these two factors [7,11].

Cryptorchidism is a well-established risk factor for developing TC, and around 5-10% of all cases are associated with cryptorchidism [12]. A meta-analysis by Lip SZ et al. showed that the relative risk of developing TC for a male with a history of cryptorchidism was 2.2 to 3.8 times higher as compared to the background population [13]. In addition, the younger the boy is operated on (orchiopexy), the lower the odds ratio (OR) for later developing testicular cancer is, with OR:1.1 for boys aged zero to nine years old at surgery, and OR:2.9 for boys aged 10-14 years old at surgery, as shown in a Danish epidemiological study [14]. All these data support the strong recommendation made by the European Association of Urology to perform orchiopexy by the age of 18 months at the latest [15].

Fertility preservation is of utmost importance in all patients with TC and especially those who suffer from bilateral testicular malignancies [16-17]. Despite that, it is estimated to be provided in only 50% of all patients being treated for TC, with sperm cryopreservation appearing to be most cost-efficient [16].

In our case, both patients had reported a history of cryptorchidism and were both diagnosed with TC at the same age, in accordance with the data already mentioned, even though the second twin sought medical advice right after his brother's diagnosis. Monozygotic twins present a 75-fold increased risk of developing TC [18]. The prognosis of testicular cancer varies among the different stages of the disease, with the majority of patients having over 95% five-year survival rates [19].

## Conclusions

Concomitant testicular cancer in monozygotic twins is a rare entity. The present case is in accordance with the data that suggest a familial risk of TC and increased risk for developing TC in patients with a history of cryptorchidism. Our clinical case adds to the necessity for a lifetime-follow up protocol in patients operated for testicular maldescent.
